# Microaxial pump-assisted Ross Procedure: Young adults with aortic valve disease and ventricular dysfunction

**DOI:** 10.1016/j.xjtc.2023.09.011

**Published:** 2023-09-18

**Authors:** Stephen M. Spindel, Jasmine Su, Christopher M. Zumwalt

**Affiliations:** a Section of Cardiothoracic Surgery, Department of Surgery, Ochsner Medical Center, New Orleans, La; b The University of Massachusetts, Amherst, Mass

**Figure:**
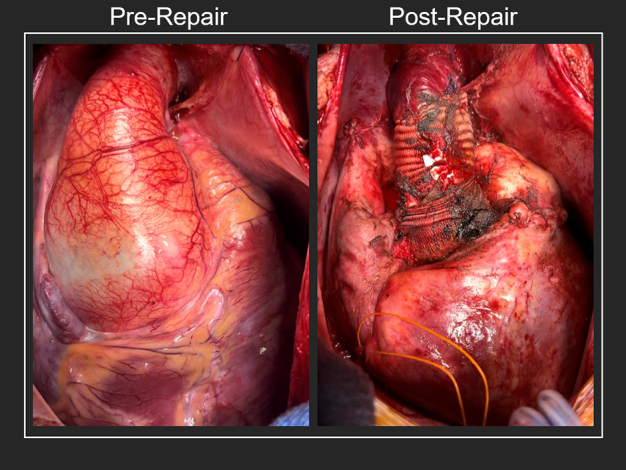
Repair of a 7.5-cm aortic root aneurysm in a patient with severe ventricular dysfunction.

**Central Message**

Aortic valve disease in young adults can present late with significant ventricular dysfunction. A 35-year-old man in cardiogenic shock underwent a Ross procedure with Impella 5.5 pump implantation.

Aortic valve disease in young adults can present late with heart failure and significant ventricular dysfunction due to their exceptional compensatory capabilities. In this case, a young man with aortic regurgitation and aortic root aneurysm presented with heart failure that progressed to cardiogenic shock was treated with an Impella 5.5 (Abiomed Inc) microaxial pump-assisted Ross procedure.

## Clinical Summary

A 35-year-old man presented with dyspnea and B-type natriuretic peptide of 2340 pg/mL was found to have severe aortic regurgitation associated with a 7.5-cm aortic root aneurysm and initial ejection fraction of 45%. The patient provided written informed consent for publication of study data; institutional review board approval was not required.

For preoperative optimization, diuresis was attempted but he quickly progressed into cardiogenic shock with altered mental status, lactic acidosis, and renal failure. With the severe aortic regurgitation, intra-aortic balloon pump was contraindicated and he was taken to the operating room for emergency surgery. The intraoperative transesophageal echocardiogram showed the ejection fraction decreased to 15% to 20% while on a moderate dose of milrinone. With these findings, an Impella 5.5 microaxial pump-assisted operation was planned. A 10-mm woven polyester graft was anastomosed to the right axillary artery and a guidewire was passed through the graft and into the aortic root. To facilitate subsequent placement of the device, this wire would later be directly inserted through the pulmonary autograft and into the ventricle during the aortic root replacement. The patient was placed on cardiopulmonary bypass, and due to persistent hyperkalemia >6 mmol/L and 0 urine output, intraoperative continuous hemodialysis was initiated. The aortic valve was trileaflet with large fenestrations, excluding repair options and valve-sparing root replacement techniques. A reinforced Ross procedure was completed with a 30-mm bulged woven polyester aortic root graft providing annular and aortic root support, plus ascending aorta replacement for sinotubular junction support. After crossclamp removal, the Impella 5.5 microaxial pump traversed the axillary graft to sit appropriately within the left ventricle. The device provided 3 L/minute flow and inotropes were at a moderate dose. Crossclamp and bypass times were 169 minutes and 238 minutes, respectively. By postoperative day 2, hemodialysis was discontinued and by day 6 the microaxial pump was removed. His recovery was uneventful and the transthoracic echocardiogram 2 weeks postoperatively showed near-normal left ventricular function (Video 1).

## Discussion

Aortic root surgery in young patients with severe left ventricular dysfunction can be challenging. Surgical options include aortic root composite graft (mechanical or biological prosthesis), valve-sparing aortic root replacement, and the Ross operation, recognizing that mechanical support such as intra-aortic balloon pump, microaxial pump placement (eg, Impella), and extracorporeal life support could be necessary. Although a mechanical prosthesis might provide longevity in a young patient, its use is not compatible with Impella pump placement and thrombosis is a concern during extracorporeal life support. A biological prosthesis in a tricenarian has reduced valvular longevity and, for Impella 5.5 placement, there is unknown risk associated with leaflet damage and early structural deterioration due to the device's persistent interaction with the leaflets.^1^ Right-sided Impella transpulmonary pump placement is commonly performed worldwide and there are no cases of pulmonary valve injury reported in the literature. With direct placement of the guidewire through the pulmonary autograft while performing a Ross operation, the risk for leaflet injury during subsequent Impella 5.5 pump placement should be reduced. Additionally, if extracorporeal life support is necessary, thrombotic complications are less of a concern with the use of a pulmonary autograft versus prosthetic valves. For long-term considerations, 15-year survival after the Ross procedure is like that of a matched US population and superior to patients with biological or mechanical aortic valve replacements. The pulmonary autograft has lower risks of reintervention and endocarditis versus biological valves and lower risks of stroke and bleeding versus mechanical valves.^2^ With these in mind, the Ross operation is ideal in a tricenarian.

The benefits of Impella pump support during cardiogenic shock have been noted in the surgical and nonsurgical settings.^3^ More recently, proactive intervention rather than reactive intervention for hemodynamic success during cardiac surgery has been on the rise. This is most notable with a planned use of Impella pump placement either a day before the operation or during the operation itself. The early results of Impella pump-assisted cardiac surgery are promising and further publications with advanced Impella designs continue to show favorable outcomes in these high-risk patients.^4^^,^^5^

Recognizing the short-term benefits of Impella pump-assisted surgery and the long-term considerations of aortic valve treatment options, the approach taken in this case highlights 1 method of tackling aortic valve disease in young patients with severe left ventricular dysfunction.

## Conflict of Interest Statement

The authors reported no conflicts of interest.

The *Journal* policy requires editors and reviewers to disclose conflicts of interest and to decline handing manuscripts for which they may have a conflict of interest. The editors and reviewers of this article have no conflicts of interest.

## Supplementary Data

Video 1 Intraoperative prebypass transesophageal echocardiogram showing aortic regurgitation with severe left ventricular dysfunction compared with the 2-week postoperative transthoracic echocardiogram showing near-normal left ventricular function. Video available at: https://www.jtcvs.org/article/S2666-2507(23)00353-X/fulltext.
