# Supplementary figures and images for: Microaxial pump-assisted Ross Procedure: Young adults with aortic valve disease and ventricular dysfunction
Source: JTCVS Tech. 2023 Sep 18;22:185–6. doi: 10.1016/j.xjtc.2023.09.011 (PMC10750847; doi:10.1016/j.xjtc.2023.09.011)

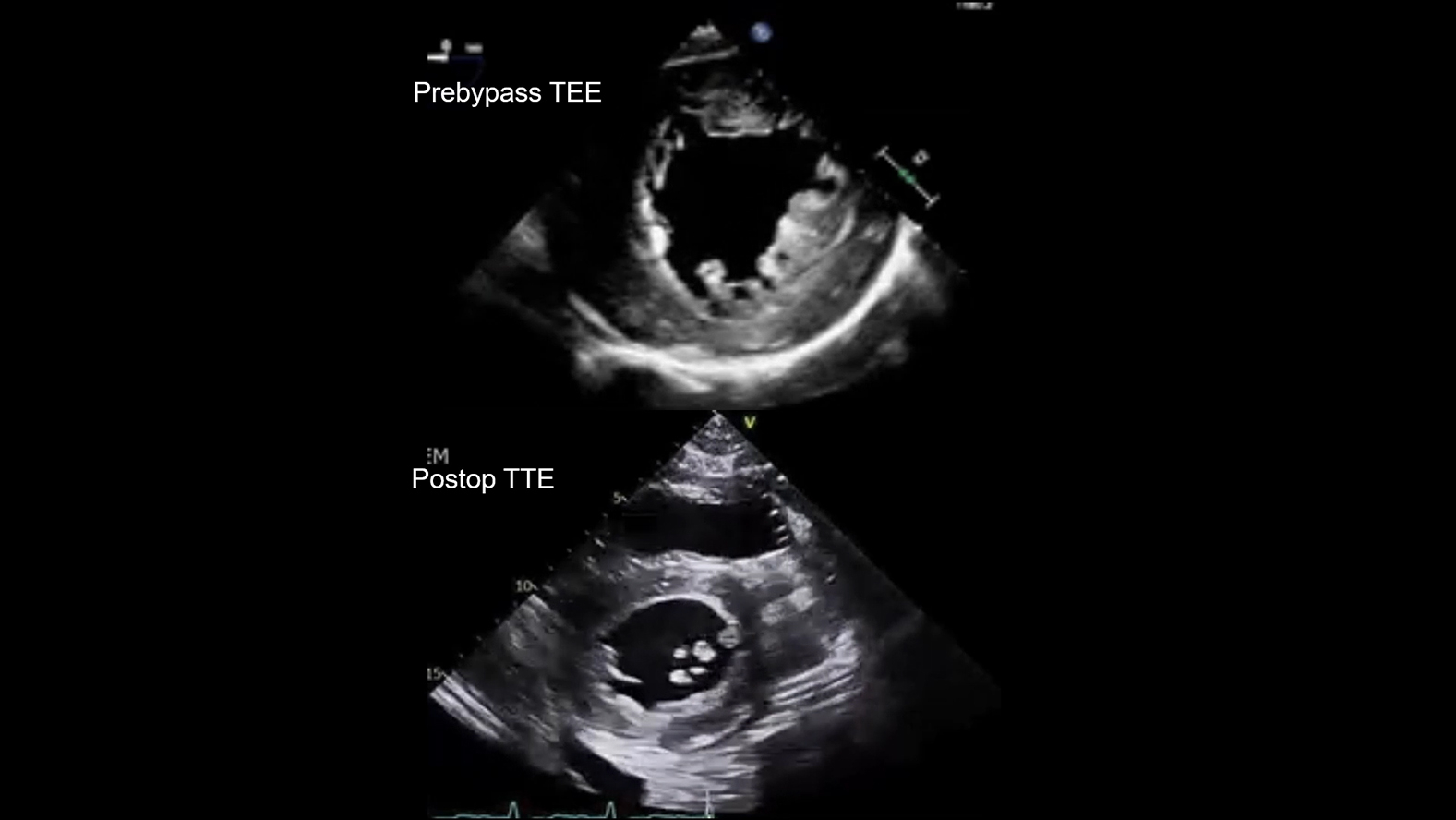

Supplement: Video 1 — Intraoperative prebypass transesophageal echocardiogram showing aortic regurgitation with severe left ventricular dysfunction compared with the 2-week postoperative transthoracic echocardiogram showing near-normal left ventricular function. Video available at: https://www.jtcvs.org/article/S2666-2507(23)00353-X/fulltext. [file fx2.jpg]
